# Large-Scale Food Fortification and Biofortification in Low- and Middle-Income Countries: A Review of Programs, Trends, Challenges, and Evidence Gaps

**DOI:** 10.1177/0379572118774229

**Published:** 2018-06-24

**Authors:** Saskia J. M. Osendarp, Homero Martinez, Greg S. Garrett, Lynnette M. Neufeld, Luz Maria De-Regil, Marieke Vossenaar, Ian Darnton-Hill

**Affiliations:** 1 Osendarp Nutrition, Berkel & Rodenrijs, the Netherlands; 2 Micronutrient Forum, Ottawa, Ontario, Canada; 3 Nutrition International, Ottawa, Ontario, Canada; 4 Global Alliance for Improved Nutrition (GAIN), Geneva, Switzerland; 5 Institute of Obesity, Nutrition and Exercise, Sydney Medical School, University of Sydney, Sydney, New South Wales, Australia; 6 Friedman School of Nutrition Science and Policy, Tufts University, MA, USA

**Keywords:** large-scale food fortification, biofortification, low- and middle-income countries, economics, compliance

## Abstract

**Background:**

Food fortification and biofortification are well-established strategies to address micronutrient deficiencies in vulnerable populations. However, the effectiveness of fortification programs is not only determined by the biological efficacy of the fortified foods but also by effective and sustainable implementation, which requires continual monitoring, quality assurance and control, and corrective measures to ensure high compliance.

**Objective:**

To provide an overview of efficacy, effectiveness, economics of food fortification and biofortification, and status of and challenges faced by large-scale food fortification programs in low- and middle-income countries (LMIC).

**Methods:**

A literature review of PubMed publications in English from 2000 to 2017, as well as gray literature, targeting nongovernmental organizations whose work focuses on this topic, complemented by national reports and a “snowball” process of citation searching. The article describes remaining technical challenges, barriers, and evidence gap and prioritizes recommendations and next steps to further accelerate progress and potential of impact.

**Results:**

The review identifies and highlights essential components of successful programs. It also points out issues that determine poor program performance, including lack of adequate monitoring and enforcement and poor compliance with standards by industry.

**Conclusions:**

In the last 17 years, large-scale food fortification initiatives have been reaching increasingly larger segments of populations in LMIC. Large-scale food fortification and biofortification should be part of other nutrition-specific and nutrition-sensitive efforts to prevent and control micronutrient deficiencies. There are remaining technical and food system challenges, especially in relation to improving coverage and quality of delivery and measuring progress of national programs.

## Introduction

Deficiencies of micronutrients (vitamins and minerals/trace elements), and the resulting negative health consequences of such deficiencies, affect over an estimated 2 billion people globally.^1^ The most common forms of micronutrient deficiencies include iron, iodine, vitamin A, zinc, and folate.^1^ The most vulnerable populations include reproductive-aged women, young children, and female adolescents, particularly in LMIC.^2,3^

Micronutrient malnutrition or “hidden hunger” has significant health and economic consequences.^4-7^ Just in LMIC, micronutrient deficiencies alone have been estimated to cost an annual gross domestic product loss of 2% to 5%^8-11^ with direct costs estimated between US$20 and US$30 billion every year.^10^ Anemia, for example, has been estimated to lead to 17% reduced lower productivity in heavy manual labor and an estimated 2.5% loss of earnings due to lower cognitive skills.^9^

The World Health Organization (WHO) and the United Nations Food and Agriculture Organization (FAO) have identified 4 main strategies for addressing micronutrient malnutrition: nutrition education leading to increased diversity and quality of diets, food fortification and biofortification, supplementation, and disease control measures.^12^ Each of these strategies has a place in reducing micronutrient malnutrition. For maximum impact, the appropriate mix of these strategies should be in place simultaneously to promote equity in access to interventions and social mechanisms that allow consumption and utilization of an adequate diet for all people in the world.^12^

Food fortification is a sound public health strategy because it can reach large segments of at-risk populations through existing food delivery systems, without requiring major changes in existing consumption patterns.^13^ Compared to other interventions, food fortification is likely to be more cost-effective, and if fortified foods are regularly consumed, there is an advantage of maintaining consistent physiological body stores of certain micronutrients used in the fortification.^12^

Large-scale food fortification programs have been in place in industrialized countries since the early 20th century and have helped to eliminate deficiency diseases in high-income countries, mainly in North America and Europe.^4,14^ More recently, food fortification has gained traction in LMIC as well, and its health impact in these countries is growing.^15^ However, the effectiveness of fortification programs is not only determined by the biological efficacy of the fortified food but also by its effective implementation, which includes among others monitoring, quality assurance (QA)/quality control (QC) followed by correction of identified issues, as well as compliance by industry with the fortifications standards.

In 2006, the WHO published guidelines for effective fortification, including the appropriate selection of food vehicles and fortificants, determining fortification concentrations, and implementing effective and sustainable food fortification programs.^12^ In September 2015, the #Future Fortified Global Summit on Food Fortification was held in Arusha, Tanzania, to discuss the state of the art on achievements and challenges in large-scale food fortification in LMIC. The Arusha Summit aimed to develop a consensus among key global stakeholders around a vision and strategy for scaling up fortification that would, in turn, contribute to the Sustainable Development Goals and beyond.^15^ The resulting “Arusha Statement on Food Fortification” (http://www.gainhealth.org/wp-content/uploads/2015/05/Arusha-Statement.pdf) summarized commitments to address remaining challenges around monitoring, compliance, and equity. It also outlined 5 critical areas needing to be addressed for immediate progress: (1) modest but new investments by governments and donors to ensure technical support and capacity, compliance, and leveraging coinvestment by the private sector; (2) improving the oversight and enforcement of food fortification standards and legislation; (3) generating more evidence to demonstrate impact and further guide fortification policy and program design; (4) more transparent accountability and global reporting; and (5) continuing advocacy for greater attention to fortification by governments.^15^

As part of the global response to the Arusha Statement, this article will provide an overview of efficacy, effectiveness, and economics of food fortification and biofortification, as well as an analysis of the overall status of large-scale food fortification programs in LMIC. The article is based on 2 detailed review reports published in the aftermath of the Arusha meeting.^16,17^ It reviews and identifies essential components of successful programs, points out issues around the frequent lack of adequate compliance, and describes remaining technical challenges, barriers, and evidence gaps. It then uses this information to prioritize recommendations and next steps, with a focus on mass fortification of staples, edible fats and oils, sugar, and condiments (including salt). In addition, it reviews the current evidence base and promise of biofortification efforts in LMIC.

## Methods

A literature review of both formal and gray literature was conducted, targeting nongovernmental organizations whose work focuses on this topic, including the Food Fortification Initiative (FFI), the Global Alliance for Improved Nutrition (GAIN), Nutrition International (NI), and the Iodine Global Network (IGN), complemented by national reports. This work was underpinned by a formal literature search, focusing on articles and reports from 2000 to 2017, in English only. Keywords used were “Fortified food*,” “Enriched food*,” “Supplemented food*,” and for Medical Subject Headings (MeSH)-Medline (via OvidSP) “Food, fortified//adverse effects.” Complementary keywords were “Government program*,” “Government sponsored program*,” “Nutritional policy,” “Government health promotion,” “Food fortification program*,” “Policymaker*,” “Health policy*,” “Mandatory program*,” and under MeSH were “Health promotion/og,” “Nutrition policy/,” “Health policy/,” “Mandatory programs/,” “Policy making,” and “Legislation, Food/.” In addition, references were added by a process of forwarding citation searching: for example, identifying relevant references of key articles such as the WHO/FAO 2006 guidelines,^12^ following them up, and then repeating the process with each article used.

## Results

### Efficacy of Food Fortification

The efficacy of food fortification has been demonstrated consistently for different micronutrients and different food vehicles.^6,12^ As a result, it is now well accepted that micronutrient fortification of foods has the potential to significantly increase serum micronutrient concentrations and reduce clinical and physiological manifestations of deficiencies.^18,19^ A systematic review of randomized and pseudorandomized controlled trials included 60 acceptable trials on iron fortification and iron biofortification and found that iron fortification of foods resulted in a significant increase in hemoglobin (0.42 g/dL, 95% confidence interval [CI]: 0.28-0.56) and serum ferritin (1.36 μg/L; 95% CI: 1.23-1.52), a reduced risk of anemia (risk ratio [RR]: 0.59; 95% CI: 0.48-0.71), and iron deficiency (RR: 0.48; 95% CI: 0.38-0.62); no effect was found on rate of infections, physical growth, or mental and motor development.^20^ The efficacy of rice fortification with iron has been demonstrated in different settings.^21-23^ In Mexico, daily consumption of iron-fortified rice 5 d/wk over a 6-month period significantly increased body iron stores and mean plasma ferritin concentration in working women between 18 and 49 years of age and improved hemoglobin concentration of women with anemia, resulting in an overall reduction of the prevalence of anemia of 80%.^21^ Fortification of wheat flour with folic acid has been widely shown to significantly improve folate status in the population, and its significant effect in reducing the risk of neural tube defects (NTDs) has been repeatedly documented and is now widely accepted.^24,25^ Efficacy of vitamin A fortification has been documented in the Philippines, where monosodium glutamate,^26^ margarine,^27^ and wheat buns are fortified with this vitamin.^28^ The efficacy of multiple micronutrient fortification has been demonstrated in studies with iron, β-carotene, and iodine-fortified biscuits in South Africa and multiple micronutrient-fortified beverages in Botswana and Tanzania.^12^

Condiments, spices, and seasonings are increasingly being used as vehicles to increase the intake of vitamins and minerals.^29^ Mandatory or market-driven condiment fortification with iron has been used with various vehicles such as soy sauce, fish sauce, salt, and bouillon cubes.^12^ Until now, most of the experience with fortification of condiments and seasonings has been with NaFe-EDTA added to soy and fish sauces in Southeast Asian countries. Other condiments, such as bouillon cubes or curry powders, are now also being fortified with iron and other vitamins and minerals.^29^ A recent systematic review has demonstrated that iron fortification of condiments is associated with increased hemoglobin, improved iron status, and reduced anemia across targeted populations.^30^

### Efficacy of Biofortification

Evidence for the efficacy of biofortified crops in improving micronutrient status has been documented in different studies, particularly for vitamin A–biofortified crops, as summarized in a review of evidence from Harvest Plus by Bouis and Saltzman.^31^ The first efficacy data on biofortification came from studies demonstrating that consumption of vitamin A–biofortified orange-fleshed sweet potato increased circulating β-carotene and had a moderate effect on vitamin A status.^31^ Biofortified provitamin A maize improved total body stores of vitamin A in 5- to 7-year-old children in Zambia and significantly improved visual function in deficient children.^32^ In Kenya, provitamin A cassava was efficacious in improving vitamin A status of schoolchildren.^33^ Iron-biofortified beans and pearl millet improved hemoglobin and total body iron stores in Rwanda and Maharashtra, India.^31^ Biofortification with other micronutrients, such as zinc-biofortified wheat or rice, has shown to be feasible and to offer bioavailable zinc, but as yet there are no efficacy trials.^31^

### Effectiveness of Food Fortification

In high-income countries, food fortification has been largely responsible for the control or elimination of several micronutrient deficiency diseases of public health significance. For instance, marked declines in the prevalence of pellagra from niacin deficiency and beriberi from thiamine deficiency were observed in the Southern United States and Canada, respectively, after voluntary and mandatory fortification of flours and bread with high-vitamin yeast.^4,14^ In the United States, mandatory large-scale fortification of enriched cereal grain products with folic acid was authorized in 1996 and fully implemented in 1998. Within 5 years, the prevalence of NTDs was dramatically reduced to around 0.66 in 1000 pregnancies or less.^34^ Fortification of cereal grain products with folic acid became mandatory in several countries soon after and has been consistently effective in reducing the prevalence of NTDs to around 0.5 × 1000 total births in countries where it has been implemented.^12,35-37^ Mandatory addition of vitamin D to milk, which started in 1965 in Canada, eliminated the widespread problem of childhood rickets.^14^ Salt iodization, in place since the 1920s in Switzerland and the United States and rapidly expanding in LMIC, has reduced goiter prevalence globally, and universal salt iodization (USI) has prevented an estimated 750 million cases of goiter in the past 25 years.^38^ After the introduction of vitamin A–fortified margarine in Denmark in 1917, the number of cases of xerophthalmia reported at Copenhagen Hospital fell by more than 90% and had been eliminated by 1918.^39,40^

Pachon et al^41^ recently published the first systematic review of evidence of the effect of flour fortification on iron status and anemia in women and children ≤15 years in LMIC. They identified only 13 large-scale flour fortification programs that collected national-level data before fortification and ≥12 months after fortification. Their analysis found that flour fortification was associated with consistent reductions in low ferritin prevalence in one-third of women but not in children. Also, there was statistically significant reduction of anemia in 4 of 12 subgroups of women and 4 of 13 subgroups of children.^41^ This study recognized several caveats, including lack of use of an adequate program design to evaluate effectiveness, a large heterogeneity in implementation, and lack of adequate use of biological markers to evaluate impact.^41,42^ The authors also mention the fact that anemia may be due to many other causes different from iron deficiency. As a way to address this last point, Barkley et al^43^ evaluated if anemia prevalence was reduced in LMIC that fortified wheat flour, alone or in combination with maize flour, with at least iron, folic acid, vitamin A, or vitamin B_12_, comparing nationally representative data before/after fortification started. In the 12 countries that had fortified, there was a 2.4% reduction in the odds of anemia prevalence, in comparison with no reduction in the odds of anemia prevalence in 20 countries that never fortified flour.^43^

In several Latin American countries, vitamin A–fortified sugar has been effective in reducing vitamin A deficiencies.^44^ In Guatemala, where the technology for fortifying sugar with vitamin A was developed, an evaluation of the fortification program carried out showed that after 12 months of implementation, low retinol levels had decreased to 5% and prevalence of human milk samples with less than 20-μg retinol/dL was reduced by 50%.^45,46^ A recent systematic evaluation of 76 studies and 41 contextual reports^15^ concluded that there is strong evidence of important and measurable improvements after food fortification in micronutrient status and health outcomes in women and children in wide geographic settings in LMIC.^15^ Fortifying with vitamin A was estimated to reduce the prevalence of deficiency in children less than 5 years from 33.3% to 25.7% globally; effectively fortifying with iron would reduce anemia by 14%; salt iodization has reduced goiter by 40% in countries such as Pakistan; and fortifying flour with folic acid has reduced NTDs by 40% to 50%.^15^

In spite of the efficacy of rice as a suitable food to be fortified, there is still only limited evidence for its effectiveness.^23^ Japan has fortified grains to add to rice before being cooked since decades ago (on the market since 1981).^47^ In Costa Rica, mandatory rice fortification with folic acid, vitamin B_1_ (thiamine), vitamin B_3_ (niacin), vitamin B_12_ (cobalamin), vitamin E, selenium, and zinc has been in place since 2001. Rice differs from other fortified food staples, such as maize or wheat, in that the grain needs to be fortified directly rather than the subproducts (eg, flour or porridge).^48,49^ The reduction of NTDs in Costa Rica is attributed to its experiences with food fortification in general, its centralized rice industry, government leadership, and private sector support.^50^ Detailed rice fortification guidelines are in development,^51,52^ and currently, a Cochrane systematic review of the fortification of rice with vitamins and minerals for addressing micronutrient malnutrition is underway.^53^

### Effectiveness of Biofortification

The primary evidence for the effectiveness of biofortification comes from provitamin A–rich orange-fleshed sweet potato in large randomized controlled trials, reaching 24 000 households in Uganda and Mozambique from 2006 to 2009.^54-56^ Introduction of orange-fleshed sweet potato in rural Uganda resulted in increased vitamin A intakes among children and women and improved vitamin A status among children. Women who got more vitamin A from the crop also had a lower likelihood of having marginal vitamin A deficiency.^55^ In addition, recent research on the health benefits of biofortified orange-fleshed sweet potato in Mozambique showed that biofortification can improve child health; consumption of biofortified orange sweet potato reduced the prevalence and duration of diarrhea in children younger than 5 years.^57^

### Economics of Food Fortification

Assessing monetary benefits across a range of countries is challenging mainly because these benefits are driven by savings in access to health care and costs of providing health care. Other costs related to the intervention may include factors such as transport. In spite of these caveats, food fortification has been recognized as one of the most cost-effective (note 1) interventions to address nutrient deficiencies in public health; for instance, top economists gathered at the Copenhagen Consensus consistenly ranked food fortification as one of the top 4 priority development interventions.^58^ Depending on the setting and micronutrient, cost-effectiveness of fortification has been estimated between $22 per disability-adjusted life year (DALY) saved for iron fortification in East Africa to $140 per DALY saved for iron fortification in Latin America,^9^ while the cost-effectiveness of fortifying staple foods with vitamin A may be as high as US$81 per DALY.

The cost–benefit (note 2) ratio of fortification depends on various other factors, such as deficiency trends, resources, food vehicle, and fortificants used. Because these costs are higher in high–middle income and high-income countries, the cost–benefit ratio of fortification tends to be higher in these countries. Therefore, the cost– benefit and cost-effectiveness will vary depending on the food vehicle and micronutrient being reviewed. Nevertheless, after a thorough review of costs and benefits, the Copenhagen Consensus proposed micronutrient fortification, particularly iron fortification of staples and salt iodization, as one of the “best-buys” among the 30 interventions they considered for addressing the 10 great challenges facing global development.^6^

Keeping in mind the previously mentioned caveat related to different health benefits due to differences in the severity and spread of a given micronutrient deficiency, in a review presented at the #Future Fortified summit, Horton et al estimated that the median benefit–cost ratio (note 3) of iron fortification in 10 countries with high levels of anemia is 8.7:1.^15^ Iodization of salt had a benefit–cost ratio of around 30:1, while for folic acid, the range extended from 11.8:1 in Chile to 30:1 in South Africa.^15^ For an annual cost of $286 million, the Copenhagen Consensus estimated the corresponding benefits would be $2.7bn (a benefit–cost ratio of 9.5:1).^5^

### Economics of Biofortification

For biofortification, the cost-effectiveness will be dependent on the crop, micronutrient, and delivery country.^31^ Cost-effectiveness data are currently available for orange-fleshed sweet potato in Uganda, where biofortification was demonstrated to cost US$15 to US$20 per DALY saved, which the World Bank considers highly cost-effective.^31,59^

Results of cost-effectiveness studies have shown that for each of the country–crop–micronutrient combinations considered, biofortification is a cost-effective intervention based on cost per DALY saved, using World Bank standards.^60^ The Copenhagen Consensus concluded that for every dollar invested in biofortification, as much as US$17 of benefits may be gained.^5^

### Overview of Large-Scale Food Fortification Programs

Large-scale food fortification refers to the production capacity (more than 50 metric tons/d), often a prerequisite for mass fortification, which refers to the reach of a fortified product. The process involves the addition at central level or point of production of 1 or more micronutrients to foods commonly consumed by the general population, such as grains, salt and condiments, sugar, or edible oil, and is usually mandated and regulated by the government sector, in response to evidence of micronutrient deficiencies or where a population, or subpopulation, may benefit. These efforts are concentrated on the organized food processing sector among large- and medium-sized industries.

Many food vehicles have been mandated for fortification with programs that have gone to scale. For example, by the end of 2017, over 140 countries implemented national USI programs, more than 90 nations had mandatory fortification programs for at least 1 kind of cereal grain (wheat, maize, or rice), and over 50 mandated the fortification of edible oils, margarine, or ghee. Sugar is fortified in a smaller number of countries. Progress on a range of indicators for large-scale food fortification programs of salt, staples, and edible oils is regularly monitored and updated by a recently launched online tool (http://fortificationdata.org/#data).^61^

**Figure 1.**
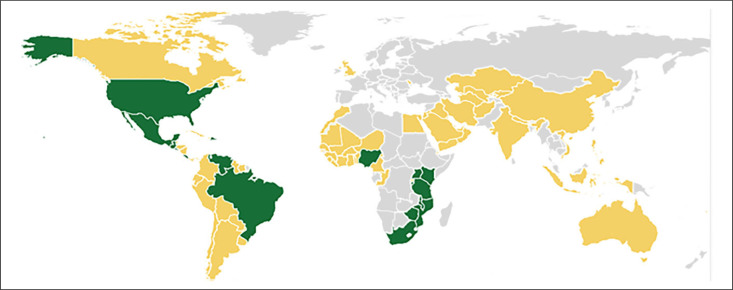
Countries with mandatory or voluntary cereal grain fortification in 2017.^61^ Yellow countries have mandatory legislation for wheat flour; green countries have mandatory or voluntary fortification of wheat and maize flour.

In 2017, there were 75 countries (plus the Indian Punjab province) with mandatory legislation to fortify wheat flour, 16 countries to fortify both wheat and maize flour, and 1 country (Rwanda) to fortify only maize flour, specifically with iron and folic acid (see Figure 1). In addition, 5 countries (Democratic Republic of Congo, Gambia, Namibia, Qatar, and United Arab Emirates) fortify at least half their industrially milled wheat flour with iron and/or folic acid through voluntary efforts.^61^ Although it is estimated that 48% of industrially milled maize flour is currently fortified,^61^ one of the main challenges to reach large segments of the population with a fortified product is that many consumers, particularly in Africa, largely consume locally produced, unprocessed (and hence unfortified) maize meal or wheat flour milled at the village level or in small-scale hammer mills.^62^ Consequently, the number of small mills without fortification technology in a country will affect whether the fortification of maize or wheat flour is a feasible option for that particular country.^62^

Of the 222 million metric tons of rice that are industrially milled each year, less than 1% are fortified with essential vitamins and minerals. Currently, 8 countries (Costa Rica, Nicaragua, Panama, Venezuela, India, Papua New Guinea, the Philippines, and the United States) have mandatory rice fortification,^61^ and Brazil, Colombia, and the Dominican Republic have large-scale nonmandatory rice fortification programs.^23^

The USI is the preferred strategy for the control of iodine deficiency disorders in most countries.^63^ Salt has been the vehicle of choice for fortification as it is consumed by nearly everyone at roughly equal amounts throughout the year and is relatively cheap/inexpensive (less than US$0.02-US$0.10 per person per year). For salt iodization, there is global information on legislation, coverage, and status (at least in children), in contrast to other food fortification programs, for which most of the information is limited to the legislation and coverage from a few countries. Salt production is often limited to a few centers, which facilitates QC, and the addition of potassium iodate or potassium iodide does not affect the taste or smell of the salt.^64^ Iodine deficiency has been considerably reduced due to iodization of salt and is now recognized as one of the great public health nutrition achievements.^64,65^

The world has moved from 110 countries iodine deficient in 1993 to now only 19 deficient countries.^38^ Nevertheless, although there is recognition of the importance of iodization of salt, some 30% of LMIC households are still not consuming iodized salt in households, with especially low coverage in some European and Central European countries, in South Asia, and some Sub-Sahara African countries.^65^

Following national-level documentation of widespread vitamin A deficiency in large sectors of the population, carried out in 1965 to 1966, sugar was legislated for fortification with vitamin A in Guatemala, Honduras, and El Salvador early in the 1970s. Sugar was chosen as the most appropriate food vehicle because of its high and stable daily consumption by the population at large, including vulnerable target groups, and its industrialized, centralized processing that facilitated adding the vitamin at minimal cost and under close supervision. Public–private partnerships were established to work toward the establishment of national programs, supported by a careful advocacy and promotion campaign. Mandatory fortification legislation was decreed in each of the countries for both domestic and industrial use. Evaluations from each of the countries showed that these programs had great success in improving vitamin A intake.^66^

**Figure 2.**
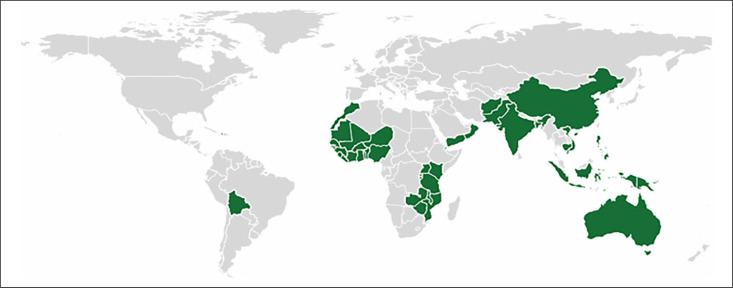
Low–middle–high income countries with mandatory or voluntary oil fortification in 2017.^61^ Green countries have mandatory or voluntary oil fortification.

Edible oils are consumed by almost everyone, usually, at uniform rates in particular regions (10-20 g/d in African countries and up to 70-90 g/capita/d in Asia),^40^ which makes them an attractive vehicle for fortification. Fortification programs for vitamin A in edible oils are currently in place in 50 countries worldwide (see Figure 2).^61^ Of these 50, well over half have mandatory fortification of margarine and/or oils, whereas 8 programs are described as “industry led” (or voluntary), 1 in which it is permitted and 7 where it was not specified.^40^ Importantly, around half of those with mandatory fortification are in LMIC.

### Components of Successful Food Fortification Programs

A recent review on the coverage and utilization of food fortification programs in 8 countries (Bangladesh, Cote d’Ivoire, India [Rajasthan], Nigeria, Senegal, South-Africa, Tanzania, and Uganda) identified some successful fortification programs, whereby the majority of the food vehicle used was fortifiable and fortified, and coverage was equitable in reaching the entire population.^67^ Programs in some other countries were identified with potential for effective fortification, largely based on very high use of a fortifiable food vehicle (note 4) by the entire population, but that potential was not currently being reached because of low compliance with fortification requirements.^68^ Four key lessons for successful programs were learned: (1) the potential for impact will depend on the appropriate choice of food fortification vehicle and on the proportion of the food vehicle consumed that is fortifiable; (2) the design of fortification programs should be informed by the magnitude and distribution of inadequate intake and deficiency and consumption of fortifiable foods, in addition, food fortification programs should be part of national micronutrient deficiency control strategies to ensure coordination with other programs; (3) effective QC of fortification levels in foods needs strengthening of capacity and resources, as well as governance and policy commitment; and (4) to ensure safe and impactful programs, periodic reviews of the assumptions related to dietary patterns that underpin food fortification are needed.^67^

In a similar review on successful staple food fortification programs in Latin America, institutional research capacity and champions of fortification, as well as private/public partnerships, were considered key features of successful and sustainable programs.^66,69^

**Figure 3.**
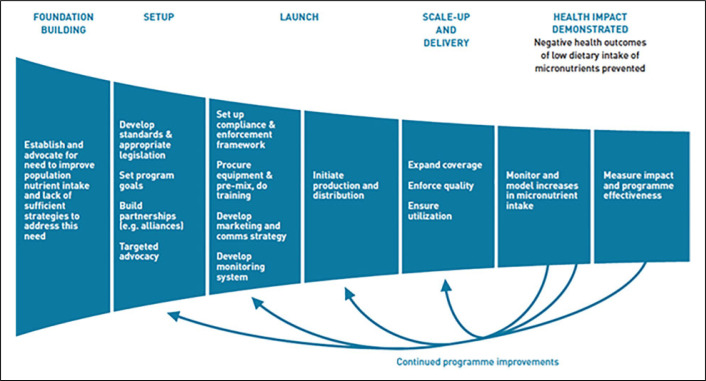
Impact model for staple food fortification.^17^

Successful programs are built on multisectoral foundations that include government, private sector, international organizations, civil society, and academia and which have worked together to generate evidence identifying the need, setting standards, ensuring legislation and alignment with national nutrition policies, ensuring QA and control throughout the manufacturing processes, and establishing strong monitoring and evaluation to ensure compliance and impact (see Figure 3).^17,66,70^

Fortification can be either mandatory or voluntary, and in both cases, appropriate standards—as set by WHO—are required to ensure impact and safety.^12^ For mandatory fortification to work, consistent and effective monitoring to ensure both QA and QC during product manufacturing and distribution, as well as consumption by the target population, are necessary.^70^ Procedures on good manufacturing practice are available through ISO and are described in the WHO/FAO guidelines.^12^ In addition, monitoring and evaluation to assess the degree to which the fortified food is actually reaching households and individuals need to be in place in order to address issues of potential for impact and utilization across different population subgroups. It is also critical for providing program planners and policymakers with the necessary information to make decisions about course correction, scaling up, or even ending of a program.^70^

### Poor Program Performance and Monitoring of Fortification Programs

A review of external QA activities in GAIN-supported staple food fortification programs in 25 countries found that the percentage of foods meeting national standards ranged from 18% to 97%, with an average around 45% to 50%.^71^ Many nonfortified foods were found to be labeled as fortified, further misleading consumers on vitamin and mineral content and contributing to a reduced health impact of fortification programs as a result of foods not appropriately fortified.^72^ It must be noted that classifying single samples as in or out of range may overestimate the level of noncompliance of fortification programs because the minimum content is highly variable depending on a range of factors. Nonetheless, 5 underlying issues were described that are leading to poor program performance and possibly poor compliance in these 25 country programs.^73^

Food laws and regulations related to monitoring, inspection, and enforcement of food fortification are often fragmented and not appropriately embedded within legal frameworks, leading to a lack of—or weak—enforcement.

Food fortification is not prioritized in food safety and QC practice and culture, especially where resources are limited. Over 80% of government respondents noted that their current funding was not sustainable over the next 5 years.

There is a perceived or real political risk in enforcing compliance with regulations. Even where resources and capacity exist, over 60% of respondents thought that regulatory agencies are often unwilling to enforce regulations due to perceived or actual resistance from interest groups.

The additional costs to industry to fortify may lead to some industries lacking appropriate internal budget and expertise to fortify appropriately while others purposely underfortify.

Regulatory monitoring agencies and consumer protection groups often do not actively protect consumers from underfortified or nonfortified foods, or fraudulent labeling, thereby misleading consumers who should be able to trust what is stated on packages in relation to vitamin and mineral content.

Given the possibility of over consumption of nutrients in groups outside the target population, identifying additional intakes and nutritional status associated with the consumption of fortified foods should be actively and consistently monitored as an integral part of any fortification program.^44,71^ In China, careful monitoring has identified counties where much of the population is likely getting too much iodine from the local water source as judged by urinary iodine levels, and in these areas, iodine is being reduced in the iodized salt distributed.^74^ Similarly, in Ireland, mandatory folic acid fortification of bread was reconsidered in 2008 because of concerns of excessive intakes due to the high intakes of voluntarily fortified foods.^75^ Using modeling techniques for fortificants, 1 study concluded that the adoption of fortification content for staple foods near the safe limit also brings into consideration the need for restricting the voluntary addition of the specific nutrient to other foods and to dietary supplements,^76^ especially where the risk of deficiency is not universal.^77^

Many national programs are currently not achieving national targets, especially in iodine “because of weak regulatory/monitoring systems” (Yusafali, MSc, personal communication, 2015). Setting up effective monitoring systems and tools for assessing QC and compliance, as well as setting up rigorous impact evaluations, requires a thorough understanding of the different pathways leading to effective coverage and impact. Insufficient budgets are often identified as constraining adequate QC and compliance.^72^

### Challenges of Large-Scale Food Fortification

Although long experience and numerous studies and reports attest to fortification’s effectiveness and feasibility,^12,78,79^ the following challenges do remain.

*Evidence gaps*. Evidence gaps remain in assessing the potential for impact on public health outcomes and how to effectively measure these. Program impact evaluations should be guided by impact pathways, prioritizing impact assessment in programs with an appropriate design and implementation to substantially increase the quality of evidence.^80^ Although the effectiveness of food fortification on nutrient intakes and nutrient status is largely established, there is still insufficient evidence of effectiveness on functional outcomes, including growth, cognitive development, morbidity, and mortality, especially in LMIC.^18,81^ This is especially an issue, as much emphasis is currently being placed on the prevention of stunting. The translation of evidence into realistic target settings for policies and programs is often lacking. In addition, changes in dietary habits over time may results in challenges, as in the case of decreasing consumption of iodized table salt in Europe,^82^ as well as opportunities, such as in the case of fortified breakfast cereals now being the dominant source of iron in UK schoolchildren.^19^

In a recent WHO/FAO Technical Consultation on scaling up rice fortification in Asia,^83^ the following research gaps on technical issues were identified: (1) assessing the stability of different micronutrients in different context-specific environments; (2) studying the nutrient–nutrient interactions, in particular related to relative bioavailability and phytate effect on iron absorption; and (3) evaluating the optimal delivery platforms for reaching the (hard to reach) target populations.

Finally, although mandated programs usually cover only registered producers, small-scale mills remain the predominant source of (iron-fortified) wheat and maize flours in many rural subsistence farming areas, while small-holding salt production units require small batch iodization. In 2015, United Nations Children’s Fund, GAIN, IGN, and NI completed a review of country experiences in small-scale salt fortification, with a smaller focus on wheat and maize fortification. The study identified number of evidence gaps and challenges for this type of food fortification, including clarity on small-scale contribution to supply; possibility of industry consolidation or quasi-consolidation in the form of cooperatives; understanding social impact; identifying incentives, models of cooperation, business plan development, and appropriate inputs for external support; establishing minimum criteria for quality; and understanding market forces and competition.^17^

*Ensuring effective coverage*. Effective coverage is defined as the proportion of the population who utilize an intervention as per intended to achieve a biological/health impact.^84^ For food fortification, this could be interpreted as the proportion of the population consuming adequately fortified food.^85^ Effective coverage is a precondition for impactful programs, along with other factors as described in this article. Challenges in reaching impact have been described from the very early days of large-scale fortification programs in the United States, and such challenges, including the choice of appropriate fortification vehicles, the use of a bioavailable fortificant, not reaching populations most likely to benefit, avoiding over consumption in nontargeted groups, and adequate monitoring of nutritional status, currently still exist in all countries.^44^ To support assessments of effective coverage in both population-based and targeted fortification programs, GAIN developed a Fortification Assessment Coverage Toolkit (FACT). An 8-country series of FACT coverage surveys were completed between 2013 and 2015 and assessed coverage (including equity aspects) of 18 identified large-scale fortification programs. Coverage varied widely by food vehicle and country, and the 2 main program bottlenecks were a poor choice of vehicle and failure to properly fortify a fortifiable vehicle (ie, the absence of adequate fortification).^68^

*Accessibility and equity*. One of the criticisms of mass fortification is that it may not be accessible to those most in need. Commercially fortified products may not be affordable for the poorest segments of societies, partly because in some countries import duties and taxes on premixes or fortification equipment drive prices up. Inequity in access to fortified foods needs to be locally researched and contextually understood, as reasons for lack of accessibility will differ within countries and within households. Programs often lack such particular understanding and do not assess intra-household food distribution practices, which are often disadvantaging women and young children within households.^86,87^ To effectively reach populations most in need, opportunities to link with, for example, social protection programs, need to be explored and better utilized.

## Conclusions and Recommendations

Food fortification is one of several evidence-based interventions that improve the overall quality of the diet, working through existing delivery systems. In addition, in recent years, biofortification has been shown as a promising, feasible, and cost-effective means of delivering micronutrients to populations who may have limited access to diverse diets and other micronutrient interventions, and efforts are underway to scale up its use to further improve global nutrition.^31^ It is important to acknowledge that, although food fortification programs are highly efficient and cost-effective, to ensure sustained impact they require continued interest and investment by governments for monitoring of delivery. In addition, investments by donors for both existing and new programs can further improve fortification’s footprint and impact. Large-scale food fortification and biofortification should be integrated into nutrition-specific and nutrition-sensitive efforts to prevent and control micronutrient deficiencies. In the past 2 decades, large-scale food fortification programs have been reaching increasingly large segments of populations in LMIC, paired with an acceleration of knowledge and guidance on large-scale fortification. Yet, a number of technical and food system challenges remain, especially in relation to improving coverage and quality of delivery and to measuring the progress of national programs. Tackling these issues in a concerted manner, as articulated in the 2015 Arusha Statement on Food Fortification,^15^ can help to further accelerate progress and potential of impact.

## Authors’ Note

SJMO conceptualized the manuscript with IDH, LMN, HM, and GG and wrote the first draft of the manuscript. IDH performed the literature review, and IDH, LMN, MV, SJMO, and HM authored the review report entitled “Large-scale food fortification: an overview of trends and challenges in Low and Middle-Income Countries in 2017” which was the basis for this manuscript. All authors provided substantial technical and editorial input in the draft versions, and reviewed and approved the final manuscript.
